# Supplementary material for: In vivo dual RNA-seq reveals that neutrophil recruitment underlies differential tissue tropism of Streptococcus pneumoniae
Source: Commun Biol. 2020 Jun 5;3:293. doi: 10.1038/s42003-020-1018-x (PMC7275033; doi:10.1038/s42003-020-1018-x)
Supplement: Supplementary file 1 — Supplementary Information [file 42003_2020_1018_MOESM1_ESM.pdf]

In vivo dual RNA-seq reveals that neutrophil recruitment underlies differential tissue tropism of *Streptococcus pneumoniae*

Supplementary Information

Supplementary Figures

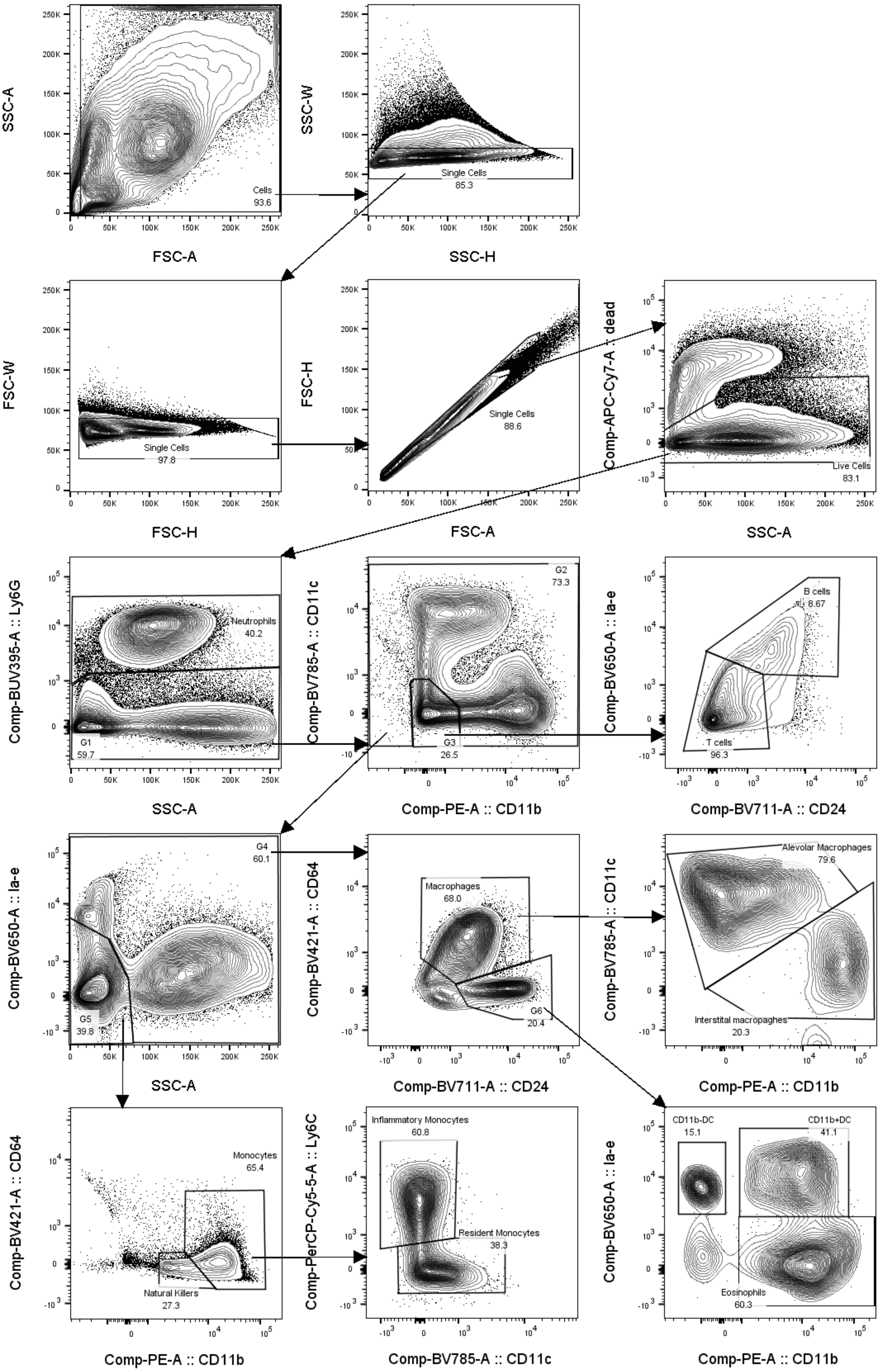

Supplementary Fig. 1 Gating strategy 1. Contour plots of windows and gating strategy used for the identification of major immune cell populations in pneumococcal infected mouse lungs. Cells were first gated for size and singularity, followed by exclusion of dead cells. Gates containing multiple cell populations other than single cell gates are numbered G1-G6. Gates containing single cell populations are labelled with the cell types that include: T cells, B cells, natural killer cells, neutrophils, eosinophils, inflammatory monocytes, resident monocytes, alveolar macrophages, interstitial macrophages, $CD11b^-$ dendritic cells ($CD11b^-$ DC), and $CD11b^+$ dendritic cells ($CD11b^+$ DC).

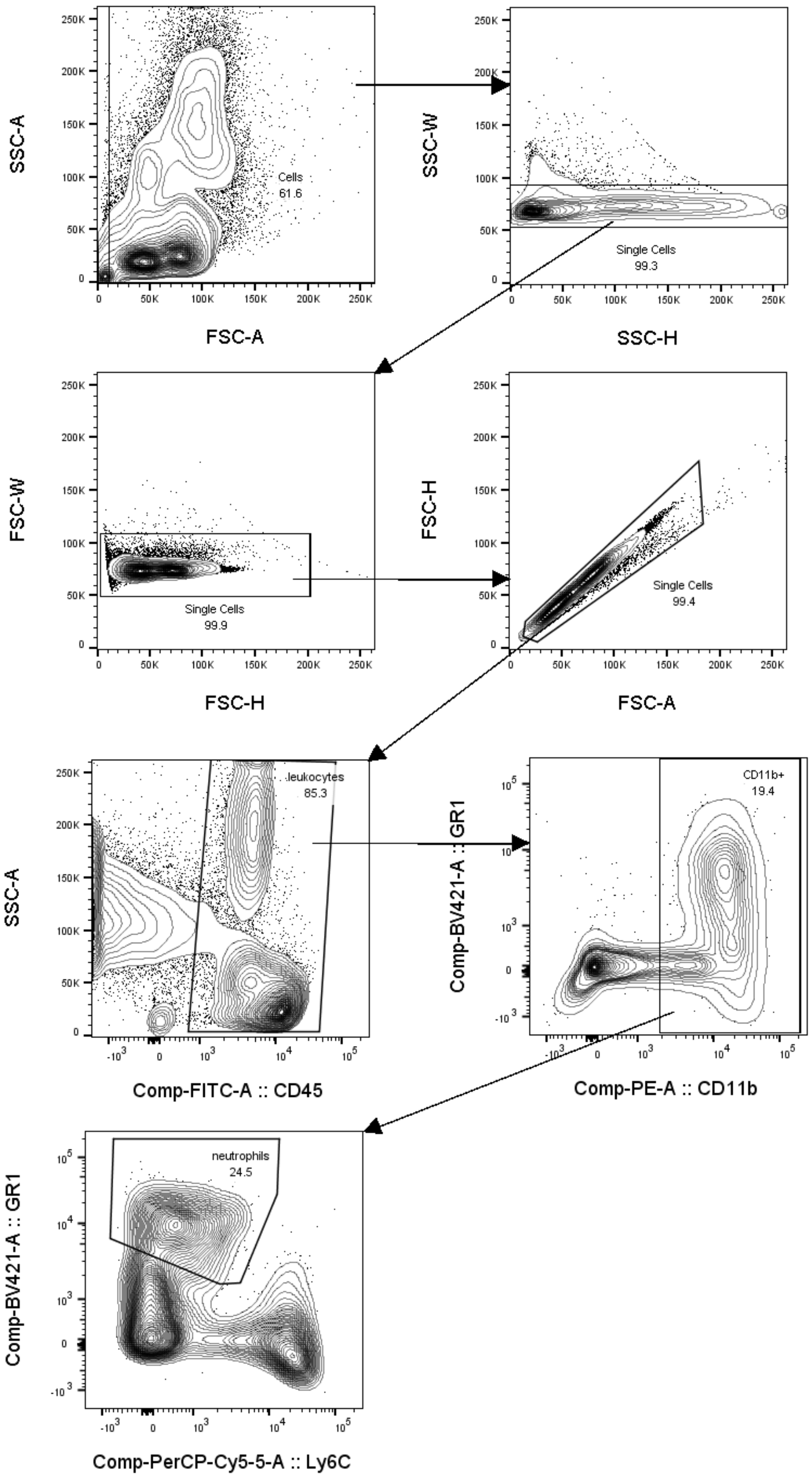

Supplementary Fig. 2 Gating strategy 2. Contour plots of windows and gating strategy used to validate anti-Ly6G mediated neutrophil depletion. Cells were first gated for size and singularity, followed by gating for leukocytes. CD11b^{+} cells were then gated for, and subsequently neutrophils were detected as GR1$^{int/hi}$ and Ly6C^{int}.

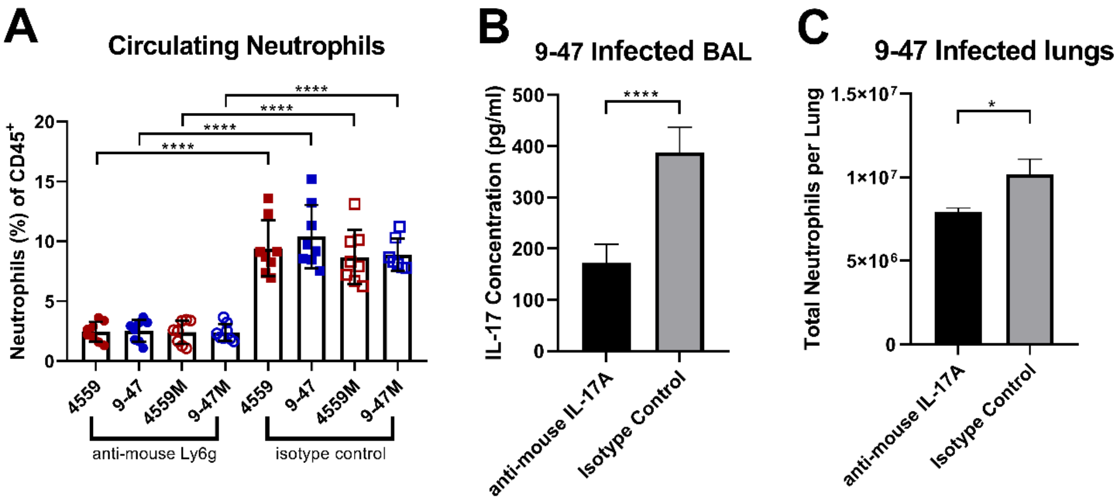

Supplementary Fig. 3 Effect of anti-mouse Ly6G and anti-mouse IL-17A treatment on neutrophil and IL-17A levels. **A** Groups of 8 mice per strain were treated with either 350 μg of rat anti-mouse Ly6G or rat IgG2a isotype control antibodies (see materials and methods). Percentage of circulating neutrophils relative to live ($CD45^+$) cells were calculated. Differences in circulating neutrophils between groups are indicated by asterisks: ****, $p < 0.0001$, by one-way ANOVA. **B & C** Groups of 3 outbred 6-week-old female Swiss (CD-1) mice were treated with either monoclonal anti-mouse IL-17A or mouse IgG1 isotype control antibodies (see materials and methods). **B** BAL was harvested 6 h post-infection and an IL-17 ELISA was performed to calculate IL-17 levels after anti-mouse IL-17A treatment. Differences in BAL IL-17 levels between groups are indicated by asterisks: ****, $p < 0.0001$, by two-tailed unpaired Student's *t*-test. **C** Lung tissue was also harvested 6 h post-infection and stained with antibodies against neutrophil surface markers (Supplementary Table 16), then analyzed by flow cytometry. Differences in lung neutrophil levels between groups are indicated by asterisks: *, $p < 0.05$, by two-tailed unpaired Student's *t*-test.

Supplementary Tables

Supplementary Table 1. Host and pneumococcal read counts of RNA isolated from murine lungs 6 h post-infection, for dual RNA-seq analysis.

Strain	Total Count	Murine Reads	Pneumococcal reads	% Murine	% Pneumococcal
9-47	46316502	46141193	175309	99.621%	0.379%
9-47	59564884	59248907	315977	99.470%	0.530%
9-47	40204707	40059925	144782	99.640%	0.360%
9-47M	30056978	29956541	100437	99.666%	0.334%
9-47M	47479606	47290932	188674	99.603%	0.397%
9-47M	48752703	48544160	208543	99.572%	0.428%
4559	40887116	40652817	234299	99.427%	0.573%
4559	42418850	42218273	200577	99.527%	0.473%
4559	36017301	35853955	163346	99.546%	0.454%
4559M	25210330	25045001	165329	99.344%	0.656%
4559M	52201495	51833174	368321	99.294%	0.706%
4559M	34141985	33825643	316342	99.073%	0.927%

Supplementary Table 2 Pneumococcal genes that were consistently up or down regulated between strains that persisted in murine lungs (4559-Blood and 9-47M) or between the strains that were cleared from the lungs (9-47-Ear and 4559M).

Gene (locus tag)	Comparison	Fold Change	padj
adhP (Sp947_00279)	9-47-Ear vs 9-47M	0.48	0.004
"	9-47-Ear vs 4559-Blood	0.32	5.38×10^{-7}
"	9-47M vs 4559M	2.39	0.00018
"	4559-Blood vs 4559M	3.53	2.23×10^{-8}
nanB (Sp947_00844)	9-47-Ear vs 9-47M	313	3.08×10^{-1}
"	9-47-Ear vs 4559-Blood	2.53	1.14×10^{-8}
"	9-47M vs 4559M	0.01	4.59×10^{-8}
glpO (Sp947_02129)	9-47-Ear vs 9-47M	5.86	1.19×10^{-37}
"	9-47-Ear vs 4559-Blood	1.89	2.26×10^{-7}
"	9-47M vs 4559M	0.25	3.55×10^{-23}

Supplementary Table 3. Oligonucleotide primers used in this study.

Primer	Sequence (5' → 3')	Reference
rafR F:	CCAGCCATTCGTGATACATA	Minhas *et al.*
rafR R:	CCTCCAGTGATTCCTAACCA	Minhas *et al.*
aga F:	AAGGTCAGAATGGTCCACAG	Minhas *et al.*
aga R:	GCTGGAAAATCAGCCATAAA	Minhas *et al.*
rafG F:	CCTATGGCAGCCTACTCCATC	Minhas *et al.*
rafG R:	GGGTCTGTGGAATCGCATAGG	Minhas *et al.*
rafK F:	GCTGGTTTACGTTCCAAGAA	Minhas *et al.*
rafK R:	GCTGGTTTACGTTCCAAGAA	Minhas *et al.*
Sp947_00054 F:	GCAAGACAGACTACGAAGCAG	This study
Sp947_00054 R:	TCCTCAATCCCATGAGCTC	This study
Sp947_00279 F:	GTGGCACTTGCGAATACTGT	This study
Sp947_00279 R:	GGATCAAGTCCGTCAGGAAC	This study
Sp947_00544 F:	CTGTTCGAGCCTCGTAACTC	This study
Sp947_00544 R:	CGTGGAAGGTGGATATTCTC	This study
Sp947_00675 F:	CCGTGTTGGTTGGAAACCAG	This study
Sp947_00675 R:	CTTGACCAGCATCACCAAGG	This study
Sp947_00841 F:	GGTTGCGTTGACTGGTAGTT	This study
Sp947_00841 R:	CCAATACCAGCTTCTGCTCC	This study
Sp947_01448 F:	ACAGCTCCAGCTATGAAGGG	This study
Sp947_01448 R:	AGACTGAGCCCCATAAGATG	This study
Sp947_01582 F:	GTCAACTGTGCAGGTCTTGC	This study
Sp947_01582 R:	GCTCCATCCTGCATATGCAT	This study
Sp947_01598 F:	GTTCGATTGCTATCGATGGT	This study
Sp947_01598 R:	CATCATATTCTTGGGTAACGC	This study
Sp947_01629 F:	CCAGTCCTTGTTGCAGTCTG	This study
Sp947_01629 R:	CGCATCAGACACAACCAACA	This study
Sp947_01798 F:	CGAGATATCGCTGCTGAGTA	This study
Sp947_01798 R:	CAAACGCTCTGTTCTGGAAC	This study
Sp947_01920 F:	TCCATGGATACCTCAACTCG	This study
Sp947_01920 R:	CTAGAGGCGTCGTATCTCGA	This study
Sp947_01951 F:	AATGGTCATTCCAGAAGCAG	This study
Sp947_01951 R:	CTTCTTGGATAAGCAGGTGTC	This study
Sp947_01955 F:	CCATGCCATGGTAGAGCTTG	This study
Sp947_01955 R:	TGGCAGCATCCATTGGAGAC	This study
Sp947_01982 F:	AGGCAAGCAGTACAGGCAAC	This study
Sp947_01982 R:	GTCCTGCTTGATTTCGACAG	This study
Sp947_02097 F:	CATTCTTGCTCCTCTCCAAG	This study
Sp947_02097 R:	GATTGATCATGAGACCTGCG	This study
ENSMUSG00000063021 F:	CTGCTTGCCTCTTCCTGACAT	This study
ENSMUSG00000063021 R:	ATTGGTCTAGGTGCAATGCTTC	This study
ENSMUSG00000068855 F:	AAGTGACGATCGCACAGGG	This study
ENSMUSG00000068855 R:	CGTGTTGAGTTTCACTTGCTCT	This study
ENSMUSG00000063954 F:	AACTACGCGGAGCGTGTGG	This study
ENSMUSG00000063954 R:	CGCGTCTTCTTGTTGTCGC	This study
ENSMUSG00000034855 F:	TAAACTCATGGCACCGGCAT	This study
ENSMUSG00000034855 R:	GGCATTTGGCAGCTTTACCC	This study
ENSMUSG00000048806 F:	GCACTGGGTGGAATGAGACT	This study
ENSMUSG00000048806 R:	GTGGAGAGCAGTTGAGGACA	This study

ENSMUSG00000074695 F:	AGCTGCTTGGGCTTCATAAC	This study
ENSMUSG00000074695 R:	CCCCTGCAATCACCTAATCC	This study
ENSMUSG00000000157 F:	CACCTGGCTCCTTGGAGAG	This study
ENSMUSG00000000157 R:	AGCCAAGTGGAAATCGTTGT	This study
ENSMUSG00000017300 F:	AGGAACTGGCTGAGTGCTTC	This study
ENSMUSG00000017300 R:	GCTCCATCTGCTCTCAGGTC	This study
ENSMUSG00000030730 F:	CCTCCCTACCTTGATGCCAG	This study
ENSMUSG00000030730 R:	GGAAGGGTCAAGGCTTCAGG	This study
ENSMUSG00000026407 F:	CATGAGGACCTGAGGTGCAG	This study
ENSMUSG00000026407 R:	CTGGTTTGACTCTGCTGGCT	This study
ENSMUSG00000026985 F:	GCCATGTCTTCTCAAAGCAAT	This study
ENSMUSG00000026985 R:	TGAACCCTGTAGTTTCTGGGAG	This study
ENSMUSG00000061928 F:	GGCACTGGCATAGCCTCATA	This study
ENSMUSG00000061928 R:	TTCCAGAGACTACCCCACCC	This study
ENSMUSG00000063130 F:	CCCTCCACGGGACTTTGTC	This study
ENSMUSG00000063130 R:	CAATGACCCCCAGCTCTACT	This study
ENSMUSG00000030046 F:	GCATGACCCTTTGCTGGTTG	This study
ENSMUSG00000030046 R:	CCAGATCCTGCTCATGGGTG	This study
ENSMUSG00000033765 F:	ACAAGCTCCAACTCGTCGTC	This study
ENSMUSG00000033765 R:	CTCCAGATGGCACAGCATCC	This study
ENSMUSG00000028001 F:	CTGCACCCGTTTCCTAACCT	This study
ENSMUSG00000028001 R:	CACATGGTCAAGTCCCTGCC	This study
ENSMUSG00000033860 F:	TGGAGAGACTCCAGGGATAC	This study
ENSMUSG00000033860 R:	GTTTGTCTGACAGCGCATGA	This study
ENSMUSG00000033831 F:	TGGACAGTCATACAGAACCGT	This study
ENSMUSG00000033831 R:	TTCACTCGCAGTCTTTACCTG	This study

Supplementary Table 4. Bacterial strains, antibodies, chemicals and commercial assays used in this study.

**Bacterial Strains**	**Source**	**Identifier**
Streptococcus pneumoniae: Clincal blood isolate capsular serotype 14, Multi Locus Sequence Type 15: 4559-Blood	Minhas *et al.*	N/A
Streptococcus pneumoniae: Clincal isolate ear capsular serotype 14, Multi Locus Sequence Type 15: 9-47-Ear	Minhas *et al.*	N/A
Streptococcus pneumoniae: Clincal isolate ear capsular serotype 14, Multi Locus Sequence Type 15: 4559M (4559-Blood containing *rafR* of 9-47-Ear)	Minhas *et al.*	N/A
Streptococcus pneumoniae: Clincal isolate ear capsular serotype 14, Multi Locus Sequence Type 15: 9-47M (947 containing *rafR* of 4559-Blood)	Minhas *et al.*	N/A
**Antibodies**	**Source**	**Identifier**
Anti-mouse/human CD11b-PE (clone M1/70)	BioLegend	Cat# 101208, RRID: AB_312791
Anti-mouse CD11c-BV786 (clone HL3)	BD Biosciences	Cat# 563735, RRID: AB_2738394
Anti-mouse CD24-BV711 (clone M1/69)	BD Biosciences	Cat# 563450, RRID: AB_2738213
Anti-mouse CD45-FITC (clone 30-F11)	BioLegend	Cat# 103107, RRID: AB_312972
Anti-mouse CD64-BV421 (clone X54-5/7.1)	BioLegend	Cat# 139309, RRID: AB_2562694
Anti-mouse Ly6C-PerCP/Cy5.5 (clone HK1.4)	BioLegend	Cat# 128011, RRID: AB_1659242
Anti-mouse Ly6G-BUV395 (clone 1A8)	BD Biosicences	Cat# 563978, RRID: AB_2716852
Anti-mouse I-A/I-E-BV650 (clone M5/114.15.2)	BD Biosicences	Cat# 563415, RRID: AB_2738192
Anti-mouse Ly6G (clone 1A8)	Bio X Cell	Cat# BE0075-1, RRID: AB_1107721
Rat IgG2A Isotype Control (clone 54447)	R and D Systems	Cat# MAB006, RRID: AB_357349
Anti-mouse Ly-6G, Ly-6C-Biotin (clone RB6-8C5)	BD Biosicences	Cat# 553125, RRID: AB_394641
Anti-mouse IL-17A (clone 17F3)	Bio X Cell	Cat# BE0173, RRID: AB_10950102
Mouse IgG1 Isotype Control (clone MOPC-21)	Bio X Cell	Cat# BE0083, RRID: AB_1107784
**Chemicals**	**Source**	**Identifier**
Zombie NIR™ Fixable Viability Kit	BioLegend	Cat# 423105
BV421 Streptavidin	BD Biosicences	Cat# 563259
Horse serum, heat inactivated	Thermo Fisher Scientific	Cat# 26050088

LAB LEMCO Nutrient Broth + 10% horse serum (serum broth)	Adelaide University Technical Services Unit	Custom Synthesis
Columbia blood agar base (dehydrated)	Thermo Scientific Oxoid	Cat# CM0331T
Defribinated horse blood	Australian Ethical Biologicals	Cat# PDHB500
Pentobarbital Sodium Anaesthetic Injection	Illium	N/A
Acid-Phenol:Chloroform, pH 4.5 (with IAA, 125:24:1)	Thermo Fisher Scientific	Cat# AM9722
SuperScript® III Platinum® One-Step qRT-PCR Kit	Thermo Fisher Scientific	Cat# 11736059
TRIzol® Reagent	Thermo Fisher Scientific	Cat# 15596026
DMEM + HEPES	Thermo Fisher Scientific	Cat # 12430054
Fetal Bovine Serum	Corning	Cat# 35076CV
Collagenase from *Clostridium histolyticum*	Sigma-Aldrich	Cat# C9891
Dnase I	Sigma-Aldrich	Cat# 11284932001
Sodium azide solution	Sigma-Aldrich	Cat# 08591
Paraformaldehyde	Sigma-Aldrich	Cat# P6148
Bovine Serum Albumin Fraction V	Sigma-Aldrich	Cat# 10735
10x RBC lysis buffer	eBioscience	Cat# 00430054
**Commercial Assays**	**Source**	**Identifier**
RNeasy Mini Kit	Qiagen	Cat# 74106
Ribo-Zero Murine rRNA depletion kit	Illumina	N/A
Ribo-Zero Gram positive bacteria rRNA depletion kit	Illumina	N/A
Stranded cDNA library preparation kit	Illumina	N/A
Mouse IL-17 DuoSet ELISA	R and D Systems	Cat# DY421-05
