# Supplementary material for: In vivo dual RNA-seq reveals that neutrophil recruitment underlies differential tissue tropism of Streptococcus pneumoniae
Source: Commun Biol. 2020 Jun 5;3:293. doi: 10.1038/s42003-020-1018-x (PMC7275033; doi:10.1038/s42003-020-1018-x)
Supplement: Supplementary file 2 — Description of Additional Supplementary Files [file 42003_2020_1018_MOESM2_ESM.pdf]

Description of Additional Supplementary Files

File Name: Supplementary Data 1
Description: Pneumococcal differential gene expression in the lungs 6 h post-infection, 9-47-Ear vs 9-47M. Genes with fold change (FC) greater than 2 and $p < 0.05$ are shown. FC values highlighted in blue = upregulated in 9-47-Ear, while values highlighted in red = upregulated in 9-47M.

File Name: Supplementary Data 2
Description: Pneumococcal differential gene expression in the lungs 6 h post-infection, 9-47-Ear vs 4559-Blood. Genes with fold change (FC) greater than 2 and $p < 0.05$ are shown. FC values highlighted in blue = upregulated in 9-47-Ear, while values highlighted in red = upregulated in 4559-Blood.

File Name: Supplementary Data 3
Description: Pneumococcal differential gene expression in the lungs 6 h post-infection, 9-47-Ear vs 4559M. Genes with fold change (FC) greater than 2 and $p < 0.05$ are shown. FC values highlighted in blue = upregulated in 9-47-Ear, while values highlighted in red = upregulated in 4559M.

File Name: Supplementary Data 4
Description: Pneumococcal differential gene expression in the lungs 6 h post-infection, 9-47M vs 4559-Blood. Genes with fold change (FC) greater than 2 and $p < 0.05$ are shown. FC values highlighted in blue = upregulated in 9-47M, while values highlighted in red = upregulated in 4559-Blood.

File Name: Supplementary Data 5
Description: Pneumococcal differential gene expression in the lungs 6 h post-infection, 9-47M vs 4559M. Genes with fold change (FC) greater than 2 and $p < 0.05$ are shown. FC values highlighted in blue = upregulated in 9-47M, while values highlighted in red = upregulated in 4559M.

File Name: Supplementary Data 6
Description: Pneumococcal differential gene expression in the lungs 6 h post-infection, 4559-Blood vs 4559M. Genes with fold change (FC) greater than 2 and $p < 0.05$ are shown. FC values highlighted in blue = upregulated in 4559-Blood, while values highlighted in red = upregulated in 4559M.

File Name: Supplementary Data 7
Description: Murine differential gene expression in the lungs 6 h post-infection, 9-47-Ear vs 9-47M. Genes with fold change (FC) greater than 1.5 and $p < 0.05$ are shown. FC values highlighted in blue = upregulated in 9-47-Ear, while values highlighted in red = upregulated in 9-47M.

File Name: Supplementary Data 8
Description: Murine differential gene expression in the lungs 6 h post-infection, 9-47-Ear vs 4559-Blood. Genes with fold change (FC) greater than 1.5 and $p < 0.05$ are shown. FC values highlighted in blue = upregulated in 9-47-Ear, while values highlighted in red = upregulated in 4559-Blood.

File Name: Supplementary Data 9
Description: Murine differential gene expression in the lungs 6 h post-infection, 9-47-Ear vs 4559M. Genes with fold change (FC) greater than 1.5 and $p < 0.05$ are shown. FC values highlighted in blue = upregulated in 9-47-Ear, while values highlighted in red = upregulated in 4559M.

File Name: Supplementary Data 10
Description: Murine differential gene expression in the lungs 6 h post-infection, 9-47M vs 4559. Genes with fold change (FC) greater than 1.5 and $p < 0.05$ are shown. FC values highlighted in blue = upregulated in 9-47M, while values highlighted in red = upregulated in 4559.

File Name: Supplementary Data 11
Description: Murine differential gene expression in the lungs 6 h post-infection, 9-47M vs 4559M. Genes with fold change (FC) greater than 1.5 and $p < 0.05$ are shown. FC values highlighted in blue = upregulated in 9-47M, while values highlighted in red = upregulated in 4559M.

File Name: Supplementary Data 12
Description: Murine differential gene expression in the lungs 6 h post-infection, 4559-Blood vs 4559M. Genes with fold change (FC) greater than 1.5 and $p < 0.05$ are shown. FC values highlighted in blue = upregulated in 4559-Blood, while values highlighted in red = upregulated in 4559M.

File Name: Supplementary Data 13
Description: Source data files for Figures 2-7.
