# Supplementary material for: In vivo dual RNA-seq reveals that neutrophil recruitment underlies differential tissue tropism of Streptococcus pneumoniae
Source: Commun Biol. 2020 Jun 5;3:293. doi: 10.1038/s42003-020-1018-x (PMC7275033; doi:10.1038/s42003-020-1018-x)
Supplement: Supplementary file 5 — Reporting Summary [file 42003_2020_1018_MOESM5_ESM.pdf]

nature research

Corresponding author(s): James Paton

Last updated by author(s): May 5, 2020

Reporting Summary

Nature Research wishes to improve the reproducibility of the work that we publish. This form provides structure for consistency and transparency in reporting. For further information on Nature Research policies, see Authors & Referees and the Editorial Policy Checklist.

Statistics

For all statistical analyses, confirm that the following items are present in the figure legend, table legend, main text, or Methods section.

n/a	Confirmed	
☐	☒	The exact sample size (*n*) for each experimental group/condition, given as a discrete number and unit of measurement
☐	☒	A statement on whether measurements were taken from distinct samples or whether the same sample was measured repeatedly
☐	☒	The statistical test(s) used AND whether they are one- or two-sided<br>*Only common tests should be described solely by name; describe more complex techniques in the Methods section.*
☐	☒	A description of all covariates tested
☐	☒	A description of any assumptions or corrections, such as tests of normality and adjustment for multiple comparisons
☐	☒	A full description of the statistical parameters including central tendency (e.g. means) or other basic estimates (e.g. regression coefficient) AND variation (e.g. standard deviation) or associated estimates of uncertainty (e.g. confidence intervals)
☐	☒	For null hypothesis testing, the test statistic (e.g. *F*, *t*, *r*) with confidence intervals, effect sizes, degrees of freedom and *P* value noted<br>*Give P values as exact values whenever suitable.*
☒	☐	For Bayesian analysis, information on the choice of priors and Markov chain Monte Carlo settings
☒	☐	For hierarchical and complex designs, identification of the appropriate level for tests and full reporting of outcomes
☒	☐	Estimates of effect sizes (e.g. Cohen's *d*, Pearson's *r*), indicating how they were calculated

Our web collection on statistics for biologists contains articles on many of the points above.

Software and code

Policy information about availability of computer code

Data collection	RNA was isoltaed from murine lungs 6 h post pneumococcal infection. RNA quality was checked using chip-based capillary electrophoresis. Samples were then simultaneously depleted from murine and pneumococcal ribosomal RNAs by dual rRNA-depletion as previously described (Aprianto et al., 2016). Stranded cDNA library preparation was performed according to the prescribed protocol (Illumina, US). Sequencing was performed for twelve samples in one lane of Illumina NextSeq 500, High Output Flowcell in 85 single end mode.<br><br>Flow cytometry data was acquired using BD FACSDiva.
Data analysis	For sequence data analysis:<br>Quality of raw libraries was checked (Andrews and Babraham Bioinformatics, 2010) (FastQC v0.11.8, Babraham Bioinformatics, UK). In order to improve the quality of alignment, we trimmed the reads (Bolger et al., 2014) using the following criteria: (i) removal of adapter sequence, if any, based on TruSeq3-SE library, (ii) removal of low quality leading and trailing nucleotides, (iii) a five-nucleotide sliding window was created for surviving reads, in which the average quality score must be above 20 and (iv) minimum remaining length must be above 50 (Trimmomatic v0.38). The quality of trimmed reads were confirmed using FastQC (Andrews and Babraham Bioinformatics, 2010).<br><br>Alignment was performed by RNA-STAR (v2.6.0a) (Dobin et al., 2013) with the following options: (i) alignIntronMax 1 and (ii) sjdbOverhang 84. The aligned reads were the summarized (featureCount v1.6.3) according to the chimeric annotation file in stranded, multimapping (-M), fractionized (--fraction) and overlapping (-O) modes (Liao et al., 2014). In order to compare gene expression between strains from ear and blood isolate backgrounds, we prepared a common pneumococcal annotation file using Mauve v20150226 (Darling et al., 2004).<br><br>For Flow cytometry analysis:<br>FlowJo software, version 10.4.1 used for gating and analysis of flow cytometry data.

GraphPad Prism 8, version 8.0.1 (GraphPad Software, La Jolla, CA, USA) used for all quantitative data analysis.

For manuscripts utilizing custom algorithms or software that are central to the research but not yet described in published literature, software must be made available to editors/reviewers. We strongly encourage code deposition in a community repository (e.g. GitHub). See the Nature Research guidelines for submitting code & software for further information.

Data

Policy information about availability of data

All manuscripts must include a data availability statement. This statement should provide the following information, where applicable:

- Accession codes, unique identifiers, or web links for publicly available datasets
- A list of figures that have associated raw data
- A description of any restrictions on data availability

The transcriptomic datasets are available in the GEO repository, accession number GSE123982.

Field-specific reporting

Please select the one below that is the best fit for your research. If you are not sure, read the appropriate sections before making your selection.

☒ Life sciences ☐ Behavioural & social sciences ☐ Ecological, evolutionary & environmental sciences

For a reference copy of the document with all sections, see nature.com/documents/nr-reporting-summary-flat.pdf

Life sciences study design

All studies must disclose on these points even when the disclosure is negative.

Sample size	Sample sizes were chosen on the basis of previous experience with the animal model employed.
Data exclusions	No data were excluded
Replication	All attempts at replication were successful. Experiments were repeated at least once and all data presented in the manuscript were successfully reproduced without exclusions.
Randomization	All mice used for experiments throughout this study were female, and all within a set age range for a given experiment (e.g. all mice were at 5-6 weeks of age at the start date of vaccination-challenge experiments). Mice were allocated randomly into experimental groups prior to any experiments commencing.
Blinding	The experiments were not performed blinded. All experiments were performed by Vikrant Minhas as part of his PhD project. Staff resources for routine experimental blinding were not available.

Reporting for specific materials, systems and methods

We require information from authors about some types of materials, experimental systems and methods used in many studies. Here, indicate whether each material, system or method listed is relevant to your study. If you are not sure if a list item applies to your research, read the appropriate section before selecting a response.

Materials & experimental systems

n/a	Involved in the study	
☐	☒	Antibodies
☒	☐	Eukaryotic cell lines
☒	☐	Palaeontology
☐	☒	Animals and other organisms
☒	☐	Human research participants
☒	☐	Clinical data

Methods

n/a	Involved in the study	
☒	☐	ChIP-seq
☐	☒	Flow cytometry
☒	☐	MRI-based neuroimaging

Antibodies

Antibodies used

Below are all the commercially available antibodies used in this study. Information is as follows: target, fluorophore, supplier, clone name, catalogue number and RRID, as found in supplementary table S14.

Anti-mouse/human CD11b-PE (clone M1/70), BioLegend, Cat# 101208, RRID: AB_312791
Anti-mouse CD11c-BV786 (clone HL3), BD Biosciences, Cat# 563735, RRID: AB_2738394
Anti-mouse CD24-BV711 (clone M1/69), BD Biosciences, Cat# 563450, RRID: AB_2738213
Anti-mouse CD45-FITC (clone 30-F11), BioLegend, Cat# 103107, RRID: AB_312972

Anti-mouse CD64-BV421 (clone X54-5/7.1), BioLegend, Cat# 139309, RRID: AB_2562694
Anti-mouse Ly6C-PerCP/Cy5.5 (clone HK1.4), BioLegend, Cat# 128011, RRID: AB_1659242
Anti-mouse Ly6G-BUV395 (clone 1A8), BD Biosicences, Cat# 563978, RRID: AB_2716852
Anti-mouse I-A/I-E-BV650 (clone M5/114.15.2), BD Biosicences, Cat# 563415, RRID: AB_2738192
Anti-mouse Ly6G (clone 1A8), Bio X Cell, Cat# BE0075-1, RRID: AB_1107721
Rat IgG2A Isotype Control (clone 54447), R and D Systems, Cat# MAB006, RRID: AB_357349
Anti-mouse Ly-6G, Ly-6C-Biotin (clone RB6-8C5), BD Biosicences, Cat# 553125, RRID: AB_394641
Anti-mouse IL-17A (clone 17F3), Bio X Cell, Cat# BE0173, RRID: AB_10950102
Mouse IgG1 Isotype Control (clone MOPC-21), Bio X Cell, Cat# BE0083, RRID: AB_1107784

Validation

All commercially available antibodies were validated specifically for the required experiment (flow cytometry) by the manufacturer (BD, Biolegend, Bio X Cell and R and D Systems). Antibodies were used only on species for which they have been validated by the vendor. Validation data are available on the manufacturer's website (BD bioscience; https://www.bdbiosciences.com/eu/reagents/research/antibodies- buffers/immunology-reagents/c/744843, Bio X Cell: https://bxcell.com/, BioLegend: https://www.biolegend.com/en-us/immunobiology and R and D systems: https://www.rndsystems.com/products/antibodies). Appropriate antibody dilutions were performed based on preliminary experiments.

Animals and other organisms

Policy information about studies involving animals; ARRIVE guidelines recommended for reporting animal research

Laboratory animals	5-6 week-old female Swiss (CD-1) mice
Wild animals	No wild animals involved
Field-collected samples	No samples collected from the field
Ethics oversight	Experiments involving animals were approved by the University of Adelaide Animal Ethics Committee

Note that full information on the approval of the study protocol must also be provided in the manuscript.

Flow Cytometry

Plots

Confirm that:

☒ The axis labels state the marker and fluorochrome used (e.g. CD4-FITC).

☒ The axis scales are clearly visible. Include numbers along axes only for bottom left plot of group (a 'group' is an analysis of identical markers).

☒ All plots are contour plots with outliers or pseudocolor plots.

☒ A numerical value for number of cells or percentage (with statistics) is provided.

Methodology

Sample preparation

For analysis of major immune cell populations in murine lungs, 6 h post pneumococcal infection:
Groups of 8 outbred 6-week-old female Swiss (CD-1) mice (32 in total) were anesthetized and challenged with the 1x10^8 cfu/ml of pneumococci. Mice were euthanized by CO2 asphyxiation at 6 h, then lungs were finely macerated in 1 mL prewarmed digestion medium (DMEM + 5% FCS, 10 mM HEPES, 2.5 mM CaCl2, 0.2 U mL−1 penicillin/gentamicin, 1 mg mL−1 collagenase IA, 30 U mL−1 DNase) and incubated at 37°C for 1 h with mixing every 20 min. Single cells were then prepared for acquisition on a BD LSRFortessa X20 flow cytometer as previously described (David et al., 2019). Single cell suspensions were filtered through 70 um filters (BD). Spleen and mLN were harvested, and single cell suspensions prepared by mechanical disruption through a 70 um filter. All tissue samples were incubated in red cell lysis buffer (155 mM NH4Cl and 170 mM Tris-HCl (pH 7.65) combined at a 9:1 ratio, with pH adjusted to 7.2) for 45 min at 37oC. Samples were thoroughly washed in PBS and kept on ice until staining.

Single cell suspensions were then pelleted in 96-well U-bottom trays (400 rcf, 2 min) at 2 x 10^6 cells/well. Cells were resuspended in Zombie Live Dead stain (1:1000 dilution in PBS, BioLegend) for 15 min at RT in the dark. All subsequent incubations were performed at 4oC. Cells were washed twice in FACS buffer (PBS + 1% BSA + 0.04% Sodium Azide), and blocked with murine y-globulin (200 ug/ml in FACs buffer) for 10 min. Cells were stained with primary antibodies CD11b-PE 1/200, CD11v-BV785 1/100, CD24-BV711 1/500, CD45-FITC 1/1000, CD64-BV421 1/100, Ly6C-PerCP-Cy5.5 1/200, Ly6g-BUV396 1/200 and ia/ie-BV650 1/200 for 15 min. Cells were then washed in Permwash (BD), followed by a PBS + 0.04% sodium azide wash prior to resuspension in 1% PFA.

For analysis of neutrophils on anti-ly6G treated murine blood samples:
200ul of blood was taken from mice after anti-ly6G or isotype control treatment. All blood samples were incubated in red cell lysis buffer (155 mM NH4Cl and 170 mM Tris-HCl (pH 7.65) combined at a 9:1 ratio, with pH adjusted to 7.2) for 45 min at 37oC. Samples were thoroughly washed in PBS and kept on ice until staining.

Single cell suspensions were then pelleted in 96-well U-bottom trays (400 rcf, 2 min) at 2 x 10^6 cells/well. Cells were resuspended in Zombie Live Dead stain (1:1000 dilution in PBS, BioLegend) for 15 min at RT in the dark. All subsequent

incubations were performed at 4oC. Cells were washed twice in FACS buffer (PBS + 1% BSA + 0.04% Sodium Azide), and blocked with murine y-globulin (200 ug/ml in FACs buffer) for 10 min. Cell were then stained with CD11b-PE (1/200), Ly6C-PerCP-Cy5.5 (1/200), CD45-FITC (1/1000), GR-1 Biotin (1/300) and Strep BV421 (1/200) for 15 min. Cells were then washed in Permwash (BD), followed by a PBS + 0.04% sodium azide wash prior to resuspension in 1% PFA.

Instrument

Data acquisition for all murine lung tissues samples were performed on a BD LSRFortessa X-20 flow cytometer.

Software

All flow cytometry data was analysed using FlowJo software, v10.4.1

Cell population abundance

6 h post pneumococcal infection, cell populations for each of the tested major immune cell populations varied according to the specific pneumococcal strain, as specified in Figure 5.

24 h after anti-Ly6G intraperitoneal administration, an average of 76.35% reduction in neutrophil populations was observed in murine blood relative to isotype controls (Supplementary Fig. 3A)

Following IL-17A depletion in mice as detailed in the materials and methods (Supplementary Fig. 3B), an intranasal challenge was performed using the pneumococcal strain 9-47. 6 h post-infection, a 22.23% reduction in neutrophil populations was observed in murine lungs relative to isotype controls (Supplementary Fig. 3C).

Gating strategy

For detection of T cells, B cells, natural killer cells, neutrophils, eosinophils, inflammatory monocytes, resident monocytes, alveolar macrophages, interstitial macrophages, CD11b- dendritic cells (CD11b- DC), and CD11b+ dendritic cells (CD11b+ DC) in murine lungs 6 h post pneumococcal infection, isolated cells were were identified by first gating on size and singularity, followed by exclusion of dead cells using the Zombie NIR™ stain (BioLegend). The specific gating strategy for each immune cell populations can be found in Supplementary Fig. 1.

For detection of neutrophils on anti-ly6G treated murine blood samples, isolated cells were first gated for size and singularity, followed by gating for leukocytes using CD45. CD11b+ cells were then gated (Supplementary Fig. 2), and subsequently neutrophils were detected as GR1int/hi and Ly6Cint.

☒ Tick this box to confirm that a figure exemplifying the gating strategy is provided in the Supplementary Information.
